# Wi-Fi-Based Location-Independent Human Activity Recognition via Meta Learning

**DOI:** 10.3390/s21082654

**Published:** 2021-04-09

**Authors:** Xue Ding, Ting Jiang, Yi Zhong, Yan Huang, Zhiwei Li

**Affiliations:** 1 School of Information and Communication Engineering, Beijing University of Posts and Telecommunications, Beijing 100876, China; dxue@bupt.edu.cn (X.D.); tjiang@bupt.edu.cn (T.J.); lzw_fire@bupt.edu.cn (Z.L.); 2 School of Information and Electronics, Beijing Institute of Technology, Beijing 100081, China; 3 Global Big Data Technologies Centre (GBDTC), School of Electrical and Data Engineering, University of Technology Sydney, Sydney, NSW 2007, Australia; Yan.Huang-3@student.uts.edu.au

**Keywords:** Wi-Fi sensing, human activity recognition, location-independent, meta learning, metric learning, few-shot learning

## Abstract

Wi-Fi-based device-free human activity recognition has recently become a vital underpinning for various emerging applications, ranging from the Internet of Things (IoT) to Human–Computer Interaction (HCI). Although this technology has been successfully demonstrated for location-dependent sensing, it relies on sufficient data samples for large-scale sensing, which is enormously labor-intensive and time-consuming. However, in real-world applications, location-independent sensing is crucial and indispensable. Therefore, how to alleviate adverse effects on recognition accuracy caused by location variations with the limited dataset is still an open question. To address this concern, we present a location-independent human activity recognition system based on Wi-Fi named WiLiMetaSensing. Specifically, we first leverage a Convolutional Neural Network and Long Short-Term Memory (CNN-LSTM) feature representation method to focus on location-independent characteristics. Then, in order to well transfer the model across different positions with limited data samples, a metric learning-based activity recognition method is proposed. Consequently, not only the generalization ability but also the transferable capability of the model would be significantly promoted. To fully validate the feasibility of the presented approach, extensive experiments have been conducted in an office with 24 testing locations. The evaluation results demonstrate that our method can achieve more than 90% in location-independent human activity recognition accuracy. More importantly, it can adapt well to the data samples with a small number of subcarriers and a low sampling rate.

## 1. Introduction

Human Activity Recognition (HAR) has been considered as an indispensable technology in many Human–Computer Interaction (HCI) applications, such as smart home, health care, security surveillance, virtual reality, and location-based services (LBS) [1,2]. Traditional human activity sensing approaches are the wearable sensor-based methods [3,4] and the camera (vision)-based methods [5,6]. While promising and widely used, these device-based approaches suffer from respective drawbacks, making them fail to be suitable for all the application scenarios. For instance, the wearable sensor-based method works only if the users are carrying the sensors, such as smartphones, smart shoes, or smartwatches with built-in inertial measurement units (IMUs), including gyroscope, accelerometer, magnetometer, etc. However, it is inconvenient for constant use. In addition, although the camera (vision)-based method could potentially achieve satisfactory accuracy, it is limited by certain shortcomings, such as privacy leakage, line-of-sight (LOS) and light conditions, etc. Moreover, both methods require dedicated devices, which are high cost. In addition, the durability of the devices is another critical factor that should be considered.

Recently, Wi-Fi-based human activity recognition has attracted extensive attention in both academia and industry, becoming one of the most popular device-free sensing (DFS) technologies [7,8]. Compared with the other wireless signals, such as Frequency Modulated Continuous Wave (FMCW) [9,10], millimeter-wave (MMW) [11,12], and Ultra Wide Band (UWB) [13,14,15,16,17], Wi-Fi possesses the most prominent and potential advantage, which is that it is ubiquitous in people’s daily lives. Leveraging the commercial off-the-shelf (COTS) devices, Wi-Fi-based human activity recognition obviates the need for additional specialized hardware. Beyond this, it also has the same merits as other wireless signals, including the capability to operate in darkness and non-line-of-sight (NLOS) situations while providing better protection of users’ privacy in the meantime. As a result, research on Wi-Fi-based human activity recognition has proliferated rapidly over the past decade [18,19,20,21].

Previous attempts involving Wi-Fi-based sensing yielded great achievements, such as E-eyes [22], CARM [23], etc. However, the major challenge referring to the generalization performance of the approaches and systems has not been fully explored and solved. For instance, when deployed in a room, the system must work well in each location rather than a specified location. Location-independent sensing is one of the most necessary generalization capabilities. It can also be regarded as the ability of a method to transfer among different locations. Note that this is a crucial factor to determine whether the technology can be commercialized. According to the principle of wireless perception, it is not difficult to find that human activities in different locations have different effects on signal transmission. Specifically, activities conducted by people in different locations will change the path of wireless signal propagation in different ways, leading to diverse multipath superimposed signals at the receiver. It is worth noting that these signals have different data distributions, which can be treated as different domains. Hence, it is clear that the human activity recognition model trained in a specific domain will not work well in the other domains. The most obvious solution is to provide abundant data for each domain to learn the characteristics of activities in the different domains. However, it is labor-intensive, time-consuming, and with poor user experience to obtain a large amount of data in practical applications. Therefore, how to utilize as few samples as possible to solve the problem of location-independent perception to achieve outstanding generalization performance is desired.

Some solutions have been proposed to solve the above problems, and remarkable progress has been made, which lays a solid foundation for realizing location-independent sensing with good generalization ability. The solutions fall into the following four categories: (1) Generate virtual data samples for each location [24], (2) Separate the activity signal from the background [25,26], (3) Extract domain-independent features [27], and (4) Domain adaptation and transfer learning. Some approaches involving other domains (such as environment, orientation, and person) can also be grouped into these four categories. However, they pay less attention to location-independent sensing [28,29,30,31]. Although the above methods promote the process of device-free human activity recognition from academic research to industrial application, there are still some limitations. WiAG [24] requires the user to hold a smartphone in hand for one of the training sample collections in order to estimate the parameters. Widar 3.0 [27] is limited by link numbers and complex parameter estimation methods. FALAR [25] benefits from its development of a new OpenWrt firmware which can get fine-grained Channel State Information (CSI) of all the 114 subcarriers, improving data resolution. Similarly, high transmission rates of the perception signal (such as 2500 packets/s in Lu et al. [26]) can also boost the resolution. As the author described by Zhou et al. [30], a low sampling rate may miss some key information, which accounts for the deterioration in the system performance. However, using shorter packets helps reduce latency and has less impacts on communication. The detailed discussions about the effect of different sampling rates on the sensing accuracy can be found in the evaluation in [27,30]. In summary, a location-independent method that can adapt to data with a small number of antennas and subcarriers as well as a small data transmission rate is required.

This work aims to realize device-free location-independent human activity recognition using as few samples as possible. It means that the model trained with the source domain data samples can perform well on the target domain with only very few data samples. We describe our task as a few-shot learning problem, improving the performance of the model in the unseen domain when its amount of available data are relatively small [32]. The task is also consistent with meta learning, whose core idea is learning to learn [33]. They have been successfully applied in a variety of fields to solve classification tasks. Inspired by the typical meta learning approach matching network, we apply the learning method obtained from the source domain to the target domain by means of metric learning [34,35]. Assuming that, although there is no stable feature that can describe a class of actions well, we can still identify its category through maximizing the inter-class variations and minimizing intra-class differences. Judging the category of a sample by calculating the distance can be regarded as a learning method. To realize location-independent sensing, we expect to learn not only the discriminative features representation specific to our task but also the distance function and metric relationships that can infer the label with a confident margin.

In this paper, we first comprehensively and visually investigate the effects of the same activity at different locations on wireless signal transmission. We also analyze the signal received in different antennas and subcarriers with different sampling rates. Moreover, we discuss how different locations affect signal transmission without any other variable influence factors by utilizing data collected from the anechoic chamber. Then, we propose a device-free location-independent human activity recognition system named WiLiMetaSensing, which is based on meta learning to enable few-shot learning sensing. Convolutional Neural Network (CNN) and Long Short-Term Memory (LSTM) are introduced for feature representation. Unlike the traditional feature extraction process for Wi-Fi signal based on LSTM, in this paper, the memory capacity of LSTM is utilized to retain the valuable information of the samples from all the activities. In addition, an attention mechanism-based metric learning method is used to learn the metric relations of the activity with the same or different categories. Finally, extensive experiments are conducted to explore the recognition performance of the proposed system. The evaluation refers to the property involving single location, mixed locations, and location-independent sensing. Unlike existing evaluations, we reduce the sampling rate, the number of subcarriers, and antennas. Experiments show that WiLiMetaSensing achieves satisfying results with robust performance in a variety of situations.

## 2. Preliminary

### 2.1. Channel State Information

The Wi-Fi-based wireless sensing principle is leveraging the influence of perceptual targets on the transmitted signal for recognition. During the transmission from the transmitter (TX) to the receiver (RX), the wireless signal would be refracted, reflected, and scattered when encountering obstacles and objects (dynamic or static), which results in the superposition of multipath signals at the receiver. In a Multiple Input Multiple Output (MIMO) and Orthogonal Frequency Division Multiplexing (OFDM)-based Wi-Fi communication system, this process can be described by fine-grained CSI. In recent years, the physical layer information of some commercial off-the-shelf (COTS) Wi-Fi devices has gradually become available, making it possible to obtain CSI directly [36]. Compared with coarse-grained Received Signal Strength Indicator (RSSI), CSI provides richer channel characteristics.

Letting *y* and *x* respectively denote the received signal and transmitting signal, the relation between *y* and *x* can be modeled as:

(1) y=Hx+N

where *H* is the channel matrix, and *N* is the noise vector. *H* completely describes the characteristics of the channel. The process of calculating the channel matrix is called channel estimation. *H* can be represented in either channel frequency response (CFR) in frequency domain or channel impulse response (CIR) in time domain. The former is given by

(2) $$∥H_{ij}\left(f_{k}\right)∥e^{j∠H_{ij}\left(f_{k}\right)},k\in \left[1,N_{S}\right],i\in \left[1,N_{t}\right],j\in \left[1,N_{r}\right]$$

where $H_{ij}\left(f_{k}\right)$ is a complex number, which denotes the CSI corresponding to the subcarrier *k* whose carrier frequency is $f_{k}$. $∥H_{ij}\left(f_{k}\right)∥$ and $∠H_{ij}\left(f_{k}\right)$ denote amplitude and phase, respectively. *i* and *j* are the index of TX and RX antennas, respectively. $N_{t}$ and $N_{r}$ stand for the number of antennas at the TX and RX, respectively. $N_{s}$ represents the number of subcarriers for each pair of transceiver antennas.

### 2.2. Data Acquisition

**Figure 1:**
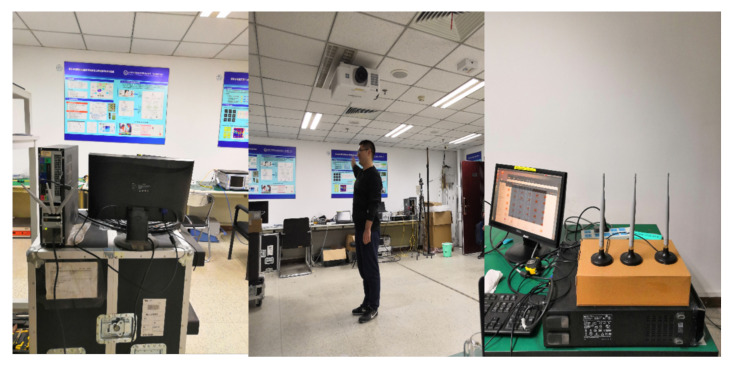
Data collection experimental scene in the office.

To thoroughly analyze the challenges in Wi-Fi-based human activity recognition and evaluate the performance of the method proposed in this paper, we built a dataset in an office environment. The data collection scene is shown in Figure 1. Specifically, Linux 802.11n CSI Tool based on Intel 5300 Network Interface Card (NIC) is leveraged to acquire the raw CSI data [36]. The TX and RX work on 802.11n and operate on a 5 GHz frequency band with a bandwidth of 20 MHz. They are both equipped with three antennas. In addition, 30 subcarriers from each TX-RX pair can be obtained. Thus, there are 3 × 3 × 30 subcarriers in total. We can only use the signal collected from part of the antennas. The data transmission rate is 200 frames/s. We can also subsample the signal measurement to verify the performance of the system at low sampling rates.

**Figure 2:**
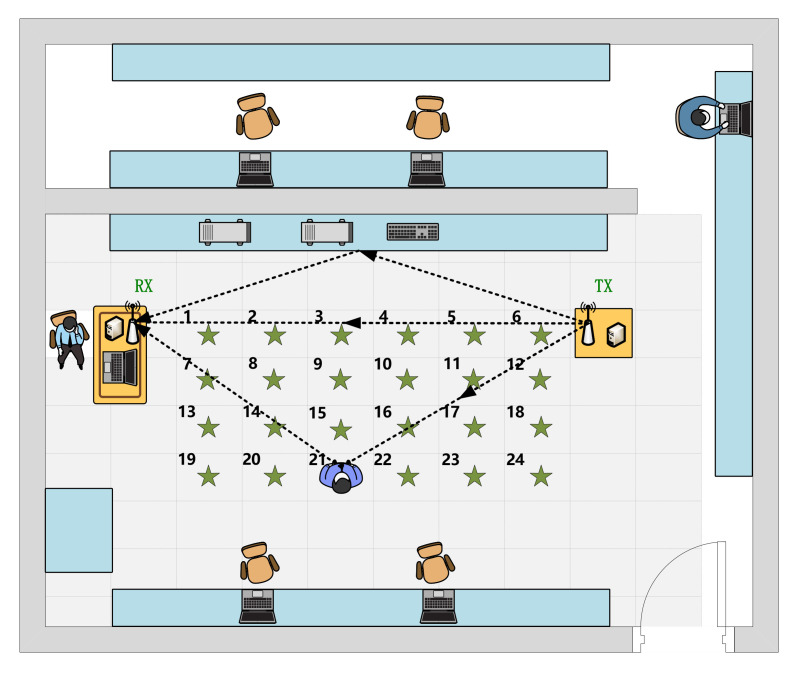
The layout of data collection locations.

Table 1 shows the predefined four activities conducted by six volunteers (five males and one female), whose ages range from 23 to 30. We collected the data in a cluttered office environment with lots of tables, chairs, and experimental facilities. The room size is approximately 6 m × 8 m. The distance between the antennas of the TX and the RX is 4 m, and the antennas were both fixed at 1.2 m above the floor. The samples are collected at 24 different locations within a region between the transceivers. The specified location layout is given in Figure 2. The distance between adjacent positions is approximately 0.6 m. We collect 50 samples for each activity at each location for each person. Since the initial sampling rate is 200 frames/s, and the actual duration of the actions is 3.5∼4 s, namely 700∼800 frames, we take 750 frames as a sample.

**Figure 3:**
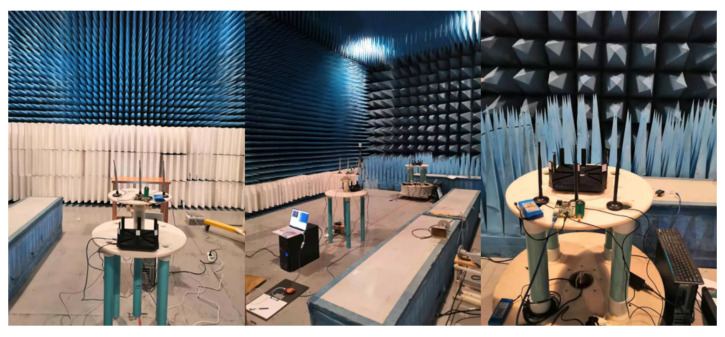
Data collection experimental scene in the anechoic chamber.

To further demonstrate the influence of activities at different locations on the transmitted signal, we also conducted some experiments in a half-wave anechoic chamber. It is a six-sided box with a shielded design, covering the electromagnetic wave absorbing material inside except for the floor. It simulates an ideal open field situation, in which the site has an infinitely large, well conductive ground plane. In a semi-anechoic chamber, since the ground is not covered with absorbent material, the reflected path will be existing, so that the signal received by the receiving antenna will be the sum of the direct and reflected path signals. More importantly, without the influence of the environment and the other same frequency wireless interference, the same activity conducted by the same person at different locations can effectively reflect the characteristics affected by the locations. The data collection scene is shown in Figure 3. Four activities in Table 1 are conducted by one person. The distance between the antennas of the TX and the RX is 3 m, and the antennas were both fixed at 1.1 m above the floor. The samples are collected at five different locations whose coordinates are (0, 0), (0.6 m, 0.6 m), (0.6 m, −0.6 m), (−0.6 m, −0.6 m), (−0.6 m, 0.6 m). (0,0) is the midpoint of the line between the TX and the RX.

### 2.3. Problem Analysis

To illustrate the issues and challenges of location-independent human activity recognition using Wi-Fi signals, we comprehensively analyze the CSI measurements involving different human activities at distinct locations collected in the office and anechoic chamber.

**Figure 4:**
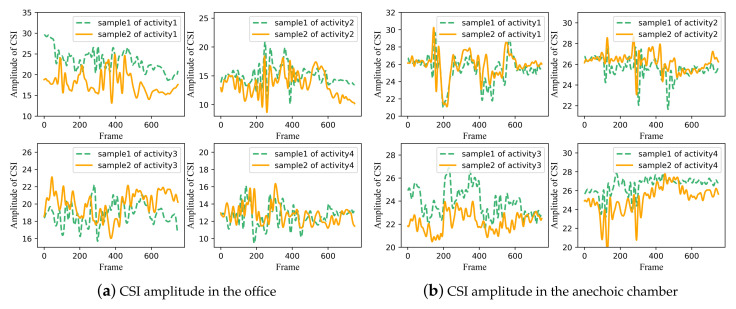
CSI amplitude of four different activities at the same location in two experimental scenes. (**a**) CSI amplitude in the office. (**b**) CSI amplitude in the anechoic chamber. Two curves in each subgraph are two samples for the same activity. The horizontal axis of each subgraph represents the frame, the ordinate of each subgraph indicates amplitude of CSI.

As shown in Figure 4, at a fixed location in both two environments, CSI amplitudes of the received signal for four different activities own different waveforms, leading to diverse characteristic patterns. Furthermore, it can be observed that the two different samples of the same activity seem to have a very similar variation tendency. These are the fundamentals of wireless sensing.

**Figure 5:**
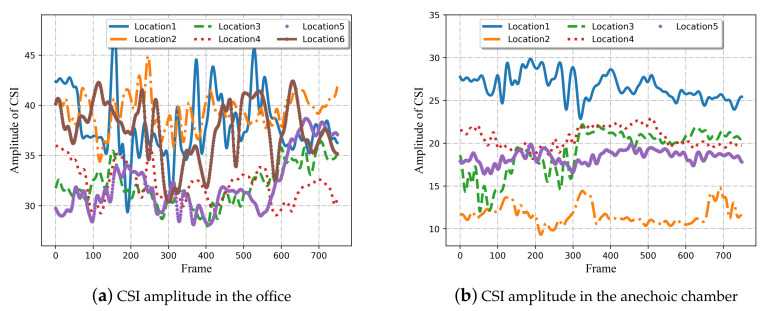
CSI amplitude of the same activity at different locations in two experimental scenes. (**a**) CSI amplitude in the office. (**b**) CSI amplitude in the anechoic chamber. Each curve in each subgraph represents an activity sample at one location.

As illustrated in Figure 5, the measured signals possess varying CSI amplitudes for the same activity at different locations. Particularly in the anechoic chamber, other variables were eliminated as far as possible except for the locations, which more clearly reflects the influence of different positions on the signal transmission. As can be seen, although it is relatively easy to identify the categories of human activities by translating the CSI patterns at a single location, it may not be possible to ensure good classification accuracy for location-independent sensing. A practicable solution is to minimize the distance of the same activity in different locations, while maximizing the distance between different actions, and apply this learned metric relationship to the target domain. For this reason, a metric learning-based approach is selected for location-independent human activity recognition.

**Figure 6:**
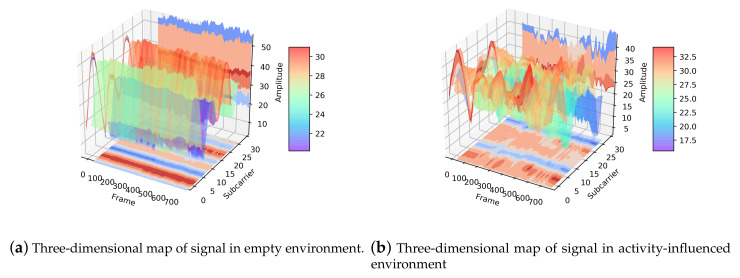
Three-dimensional map of the signal in empty environment and activity-influenced environment. (**a**) Three-dimensional map of signal in empty environment. (**b**) Three-dimensional map of signal in activity-influenced environment. The three coordinate axes are frame-axis, subcarrier-axis, and amplitude-axis, respectively. The three-dimensional waveform can be mapped to three planes, including the planes parallel to the subcarrier-axis and frame-axis, and perpendicular to the amplitude-axis.

In order to further explore the influence of activities on signal transmission, we illustrate the distinction of the signal between the empty environment and the activity-influenced environment. The three-dimensional maps of the signal are shown in Figure 6, which indicate that the fluctuation of the signal in an empty environment and activity-influenced environment. Each point on the stereogram represents the amplitude of signal corresponding to the frame and subcarrier. From the figure, we can see a higher level of chaos in the three-dimensional waveform of the activity-influenced environment than the empty environment.

**Figure 7:**
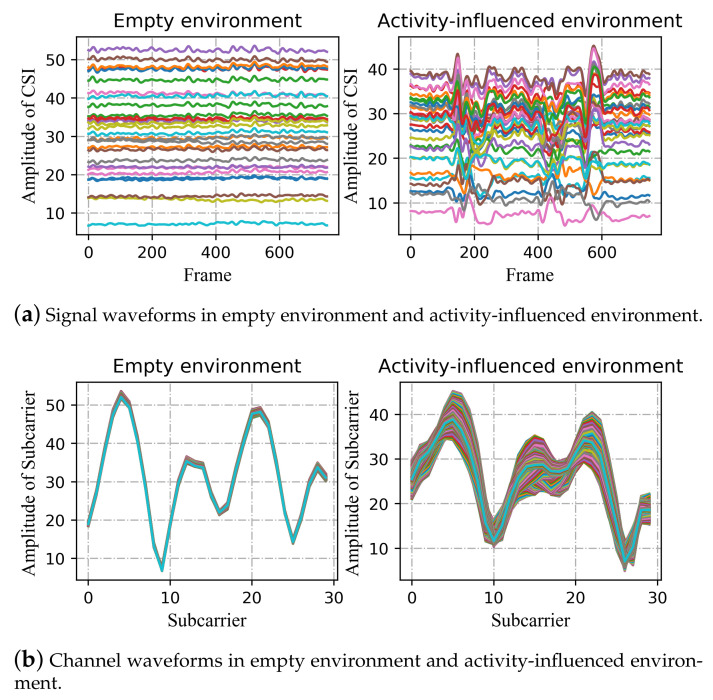
Two-dimensional map of signal in empty environment and activity-influenced environment. (**a**) The horizontal axis represents the frame/packet, the ordinate indicates the amplitude of CSI. Each curve in the figure represents one of the 30 subcarriers; (**b**) the horizontal axis represents the subcarrier index, the ordinate indicates the amplitude of the subcarriers. Each curve in the figure represents one of the 750 curves, which illustrate the amplitude change of each subcarrier within 3.5 s (The sampling rate is 200 frames/s).

To demonstrate the difference more clearly, Figure 7 shows the two-dimensional maps corresponding to the two vertical planes in Figure 6. As can be seen in Figure 7a, compared with the activity-influenced environment, the amplitude of each subcarrier is almost constant in the empty environment. In other words, the signal waveform changes smoothly with time when there is no human activity interference, while it changes obviously when the signal transmission is affected by human activity. In addition, the activity has a great influence on some subcarriers and a relatively small influence on others.

In Figure 7b, we name the curves channel waveforms, which could reflect the channel state to some extent, revealing the states of each subcarrier. The curve will change with the influence of the activity and the surrounding environment, such as other signal sources, interior layout, and furnishings, especially obstacles on the line-of-sight path. In the left figure, the amplitude of each subcarrier is almost unchanged within 3.5 s, while, in the right figure, the amplitude of each subcarrier varies to different degrees. In each environment, there is a basic channel waveform describing the channel situation (shown as the subgraph on the left of Figure 7b). After being affected by human activity, the curve generates an additional perturbation based on the basic waveform (shown as the subgraph on the right side of Figure 7b). The thickness of the whole curve represents the fluctuation degree of CSI subcarriers, which shows the extent to which human activity and the surrounding environment affect the transmission of signals. Therefore, we should pay more attention to the added activity-related changes. Deep learning methods can be used to extract action-specified characteristics.

**Figure 8:**
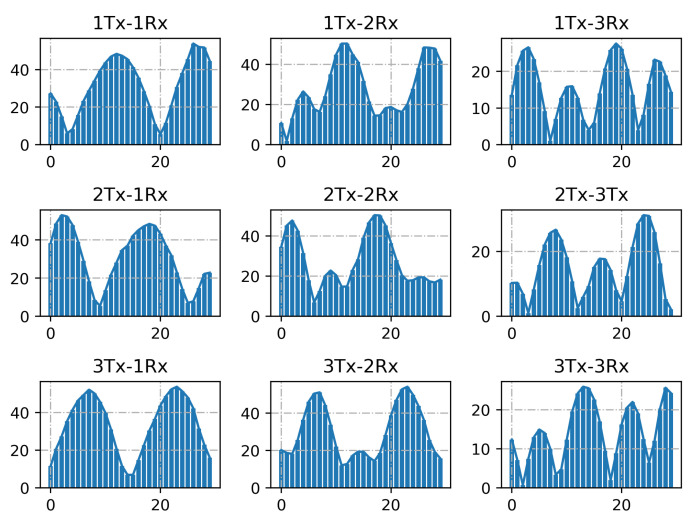
Amplitude of subcarrier of nine TX-RX antenna pairs.

We also investigate the CSI measurements in different TX-RX antenna pairs. As shown in Figure 8, we can see it intuitively, the three subgraphs of each row vary largely, and the three subgraphs of each column are similar in amplitude changes, with a horizontal shift, which can be explained by the phase shift caused by the delay of different transmitting antennas arriving at the receiving antenna. Therefore, the information carried by 1 × 3 × 30 subcarriers from one transmit antenna and three receive antennas is enough for a sample description. Although more subcarriers cover richer information, it is more desirable to extract sufficient activity characteristics from only one transceiver antenna pair, which can effectively reduce computing costs and obviate the need for the number of antennas. In this paper, we hope that the proposed method can be applied to data samples with a small number of antennas and subcarriers.

**Figure 9:**
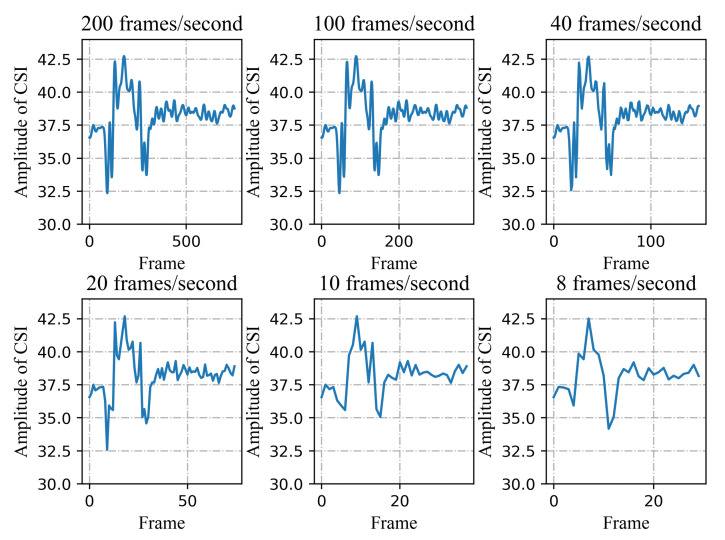
CSI Amplitude of the human activity with different sampling rates.

In this part, we study the signal affected by human activity with different sampling rates. As shown in Figure 9, as the sampling rate decreases, the signal becomes smoother. It may remove some of the noise, but, more importantly, it will lose some of the details referring to the activity. In this paper, while realizing the location-independent human activity recognition, we try our best to ensure the sensing performance of the data samples with a small sampling rate.

## 3. WiLiMetaSensing

In this section, we provide a detailed introduction to the proposed WiLiMetaSensing system. We first present the system overview. Then, a CNN-LSTM-based feature representation method is described. Finally, an attention mechanism enhanced metric learning-based human activity recognition method is presented.

### 3.1. System Overview

**Figure 10:**
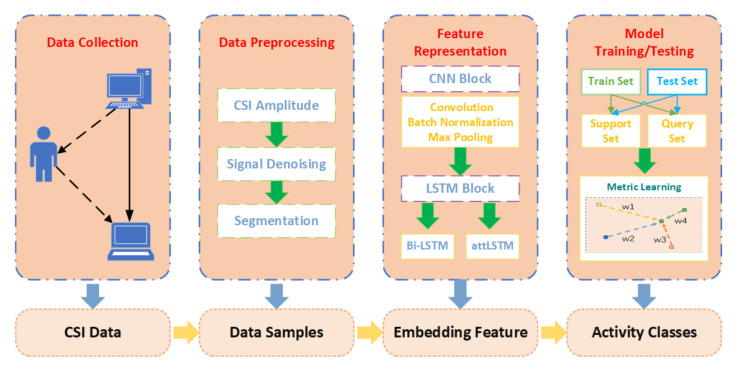
The workflow of WiLiMetaSensing.

The workflow of the location-independent human activity recognition system WiLiMetaSensing is shown in Figure 10, which mainly consists of four parts, including data collection, data preprocessing, feature representation, and model training/testing. In the data collection phase, we collect the raw CSI measurements, which describe the changes in the environment. In the data preprocessing step, the amplitude is calculated by the raw complex CSI. Due to the noisy raw data, a 5-order lowpass Butterworth filter is utilized for denoising. Beyond that, the collected data are divided into samples with the size of time × subcarrier, which indicates the number of frames corresponding to an activity multiplied by the number of subcarriers. Then, we map the data samples to high dimensional embedding space to fulfill the feature representation through CNN and LSTM. Finally, in order to achieve location-independent perception with as few samples as possible, regarding a few-shot learning problem, the human activity perceptive method based on metric learning is proposed. Subsequently, we will introduce the system in detail.

### 3.2. CNN-LSTM-Based Feature Representation

**Figure 11:**
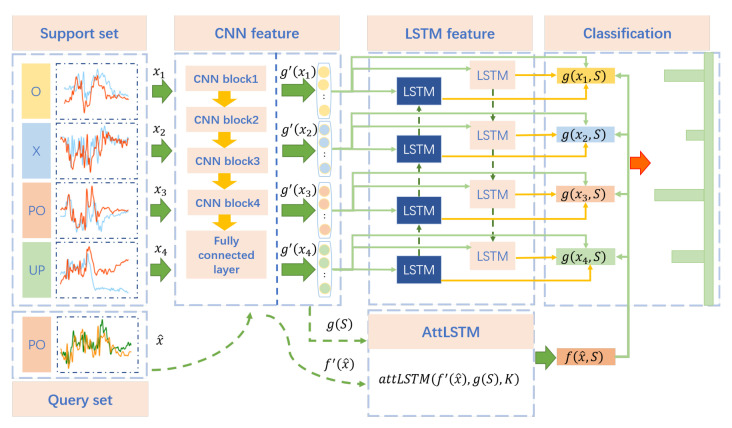
The architectures of human activity recognition method.

In this section, in order to extract activity-specified and location-independent features from input samples for few-shot learning, deep learning methods, including CNN and LSTM, are introduced for feature representation shown as Figure 11. Following the learning strategy of meta learning, the data samples are divided into two parts, including the support set and the query set with the same data selection strategy, which will be presented in detail in the next section.

We use $x_{i}$ and $\hat{x}$ to denote the samples from the above two sets. $S=\left\{x_{i}\right\},i\in 1,\ldots ,n\times k$ indicates the support set which is made up of samples from *n* categories, and *k* samples for each class. $g\left(x_{i},S\right)$ and $f\left(\hat{x},S\right)$ are modeled to achieve feature representation of $x_{i}$ and $\hat{x}$ fully conditioned on the support set, respectively.

The feature embedding function $g\left(x_{i},S\right)$ for each sample $x_{i}$ can be expressed as:

(3) $$g^{\prime }\left(x_{i}\right)=CNN\left(x_{i}\right)$$

(4) $$\vec{h_{i}},\vec{c_{i}}=\vec{LSTM}\left(g^{\prime }\left(x_{i}\right),{\vec{h}}_{i-1},{\vec{c}}_{i-1}\right)$$

(5) $$\overset{\leftarrow }{h_{i}},\overset{\leftarrow }{c_{i}}=\overset{\leftarrow }{LSTM}\left(g^{\prime }\left(x_{i}\right),{\overset{\leftarrow }{h}}_{i-1},{\overset{\leftarrow }{c}}_{i-1}\right)$$

(6) $$g\left(x_{i},S\right)=\vec{h_{i}}⊕\overset{\leftarrow }{h_{i}}+g^{\prime }\left(x_{i}\right)$$

The samples are first mapped to high-dimensional embedding space through CNN to capture the feature in subcarrier and time dimensions. Specifically, the embedding model is made up of a cascade of blocks, each including a convolutional layer, a batch normalization layer, and a MaxPooling layer, followed by a fully-connected layer. The activation function is a rectified linear unit (ReLU).

**Figure 12:**
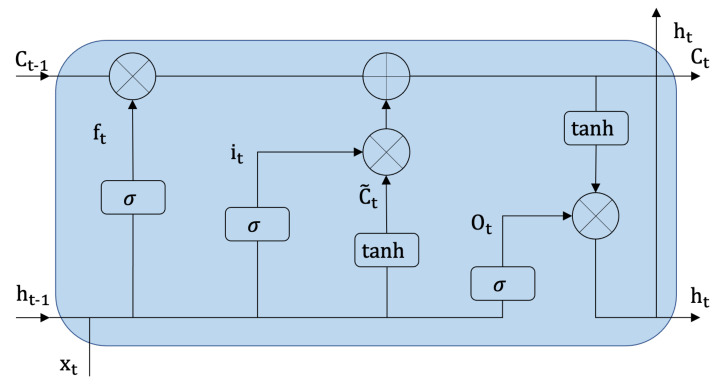
The structure of the LSTM cell.

The samples embedded by CNN form a sequence, which serves as the input of bidirectional long short-term memory (Bi-LSTM). It consists of a forward propagation LSTM and a backward propagation LSTM. The basic structure of LSTM is shown in Figure 12, which consists of three control gates, including an input gate $i_{t}$, a forget gate $f_{t}$, an output gate $o_{t}$. In addition, a memory cell $c_{t}$ and a hidden unit $h_{t}$ are also significant components. With the current input $x_{t}$, the hidden state $h_{t-1}$, and cell state $c_{t-1}$ at time t−1, the LSTM parameters at timestep *t* can be calculated as follows:

(7) $$f_{t}=\sigma \left(W_{f}\left[h_{t-1},x_{t}\right]+b_{f}\right)$$

(8) $$i_{t}=\sigma \left(W_{i}\left[h_{t-1},x_{t}\right]+b_{i}\right)$$

(9) $$o_{t}=\sigma \left(W_{o}\left[h_{t-1},x_{t}\right]+b_{o}\right)$$

(10) $${\tilde{C}}_{t}=\tanh\left(W_{c}\left[h_{t-1},x_{t}\right]+b_{C}\right)$$

(11) $$C_{t}=f_{t}\times C_{t-1}+i_{t}\times {\tilde{C}}_{t}$$

(12) $$h_{t}=o_{t}\times \tanh\left(C_{t}\right)$$

where $W_{f},W_{i},W_{o}$ are the weight and $b_{f},b_{i},b_{o}$ are the bias of the three gates. σ and tanh denote sigmoid and hyperbolic tangent activation functions, respectively. × stands for the element-wise multiplication.

Through the forget gate, the previous memory cell can be selectively forgotten. The input gate controls the current input, while the output gate determines how the memory unit is converted to a hidden unit. However, the LSTM network processes the sequential data in one direction resulting in only partial categories of features that can be utilized. Therefore, the Bi-LSTM is leveraged to merge the information from two directions of the sequence. The final hidden vector of the Bi-LSTM at the t−th moment can be expressed as:

(13) $$h_{t}={\vec{h}}_{t}⊕{\overset{\leftarrow }{\;h}}_{t}$$

where ⊕ is the concatenation operation, ${\vec{h}}_{t}$ and ${\overset{\leftarrow }{h}}_{t}$ are the outputs (hidden vector) of the forward LSTM and the backward LSTM, respectively.

Through the above CNN-LSTM feature representation, we aim to leverage the common characteristics of different activities to calibrate the high-dimensional embedding of each sample. In other words, in the feature representation of each class sample, the information of other class samples can be used. As we all know, the received CSI measurements contain not only dynamic activity information but also static environment information and varying location information. Therefore, there are some common features about the background for different samples in each category. We hope that the model can learn and memorize the common characteristics of different types of activities, as well as the distinct information of different categories. The distinct information can be utilized to increase the distance of inter-class, and reduce the distance of intra-class.

The embedding function $f\left(\hat{x},S\right)$ for a query sample $\hat{x}$ is defined as follows:

(14) $$f\left(\hat{x},S\right)=attLSTM\left(f^{\prime }\left(\hat{x}\right),g\left(S\right),K\right)$$

where $f^{\prime }$ is a neural network, the same as $g^{\prime }$. *K* denotes the number of “processing” steps following work from Vinyals et al. [37]. $g\left(S\right)$ represents the embedding function *g* applied to each element $x_{i}$ from the set *S*. Thus, the state after *k* processing steps is as follows:

(15) $${\hat{h}}_{k},c_{k}=LSTM\left(f^{\prime }\left(\hat{x}\right),\left[h_{k-1},r_{k-1}\right],c_{k-1}\right)$$

(16) $$h_{k}={\hat{h}}_{k}+f^{\prime }\left(\hat{x}\right)$$

(17) $$r_{k-1}=\sum_{i=1}^{\left|S\right|}a\left(h_{k-1},g\left(x_{i}\right)\right)g\left(x_{i}\right)$$

(18) $$a\left(h_{k-1},g\left(x_{i}\right)\right)=soft\max\left(h_{k-1}^{T}g\left(x_{i}\right)\right)$$

Noting that the $LSTM\left(x,h,c\right)$ in both *g* and *f* follows the same LSTM implementation defined by Sutskever et al. [38].

### 3.3. Metric Learning-Based Human Activity Recognition

Our location-independent activity recognition task can be described as a few-shot learning problem and a meta learning task. Meta learning trains the model from a large number of tasks and learns faster on new tasks with a small amount of data. Unlike the traditional meta learning and few-shot learning methods, which apply the model learned from some classes (source domain) to the other new classes (target domain) with very few samples from the new classes, our work is intended to utilize the model to the data with the same label, but with different data distribution.

Meta learning includes training process and testing process, which is called meta-training and meta-testing. In our task, samples in part of locations are selected as the source domain data, while samples from other locations are the target domain data. Both the source domain data and the target domain data are classified into the support set and query set with the same data set selection strategy.

Assuming that there is a source domain sample set *S* with *n* classes, and a target domain set *T* with the same *n* classes. We randomly select support sets $S^{\prime }={\left\{\left(x_{i},y_{i}\right)\right\}}_{i=1}^{n\times m}$ and $T^{\prime }={\left\{\left(x_{i},y_{i}\right)\right\}}_{i=1}^{n\times k}$, query sets $S^{\prime \prime }={\left\{\left(\hat{x},\hat{y}\right)\right\}}_{i=1}^{n\times l}$ and $T^{\prime \prime }={\left\{\left(\hat{x},\hat{y}\right)\right\}}_{i=1}^{n\times t}$ from *S* and *T* datasets. *m* and *l*, *k*, and *t* are the number of samples picked from each class of source domain and target domain, respectively. This is the so-called *k*-shot learning. More precisely, leveraging the support set $S^{\prime }$ from the source domain, we learn a function which can map test samples $\hat{x}$ from $S^{\prime \prime }$ to a probability distribution $P\left(\hat{y}|\hat{x},S^{\prime }\right)$ over outputs $\hat{y}$. *P* is a probability distribution parameterized by a CNN-LSTM feature representation neural network and a classifier. In the target domain, when a new support set $T^{\prime }$ is given, we can simply use the function *P* to make a prediction $P\left(\hat{y}|\hat{x},T^{\prime }\right)$ for each test sample $\hat{x}$ from $T^{\prime \prime }$. In short, we predict the label $\hat{y}$ for the unseen sample $\hat{x}$ and a support set $S^{\prime }$ can be expressed as:

(19) $$\hat{y}=\arg\max_{y}P\left(y|\hat{x},S^{\prime }\right)$$

A simple method to predict $\hat{y}$ is calculating a linear combination of the labels in the support set as follows:

(20) $$\hat{y}=\sum_{i=1}^{N}a\left(\hat{x},x_{i}\right)y_{i}$$

where *a* is an attention mechanism which is shown as:

(21) $$a\left(\hat{x},x_{i}\right)=\frac{e^{c\left(f\left(\hat{x}\right),g\left(x_{i}\right)\right)}}{\sum_{j=1}^{N}e^{c\left(f\left(\hat{x}\right),g\left(x_{j}\right)\right)}}$$

It is softmax over the cosine similarity *c* of the embedding functions *f* and *g*, which are the feature representation neural network. In addition, the cosine similarity is calculated as:

(22) $$c\left(f\left(\cdot \right),g\left(\cdot \right)\right)=\cos\left(f\left(\cdot \right),g\left(\cdot \right)\right)=\frac{f\left(\cdot \right)\cdot g\left(\cdot \right)}{∥f\left(\cdot \right)∥∥g\left(\cdot \right)∥}$$

The training procedure is an episode-based training, which is a form of meta-learning, learning to learn from a given support set to minimize a loss over a batch. More specifically, we define a task *T* as a distribution over possible label sets *L* (four activities in our experiment). To form an “episode” to compute gradients and update our model, we first sample *L* from *T* (e.g., *L* could be the label set X, O, PO, UP). We then use *L* to sample the support set *S* and a batch *B* (i.e., both *S* and *B* are labelled examples of X, O, PO, UP). The network is then trained to minimize the error predicting the labels in the batch *B* conditioned on the support set *S*. More precisely, the training objective is as follows:

(23) $$\theta =\arg\max_{\theta }E_{L∼T}\left[E_{S∼L,B∼L}\left[\sum_{\left(x,y\right)\in B}\log P_{\theta }\left(y|x,S\right)\right]\right]$$

where θ represents the parameters of the embedding function *f* and *g*.

## 4. Evaluation

In this section, we evaluate the performance of the proposed WiLiMetaSensing system through extensive experiments. The evaluation contains the following three parts. Firstly, we explore the feasibility and effectiveness of our system. Then, we investigate the system Modules. Finally, the robustness of the system is discussed by demonstrating the influence of different data samples.

### 4.1. Experiment Setup

We first evaluate the performance of our sensing method in a traditional way, including the single location sensing and the mixed locations sensing. In addition, we validate the effectiveness of location-independent sensing. There are 50 samples for each activity at each location for each person, 60% of which are randomly selected as the training set, 20% as the validation set, and the rest as the testing set. For single location sensing, we train and test at the same location. For mixed locations sensing, we apply the activities of all the locations for training and testing. For location-independent sensing, we show the overall average accuracy with four locations for training and 24 locations for testing. In this section, we show the overall accuracy for one-shot learning using the samples with 200 frames/s sampling rate, which lasts for 3.5 s, and 90 subcarriers. According to the training strategy of meta learning method, when we test for *k*-shot learning, we set the number of samples in each category of the support set as *k* for the testing sets. We set the support set of training and validation sets the same as the testing sets.

Specifically, the CNN embedding module consists of four CNN blocks, each including a convolutional layer, a batch normalization layer, and a 2×2 max-pooling layer, followed by a fully-connected layer with 64 neurons. In addition, 64 filters with the kernel size 3×3 are used. In the Bi-LSTM embedding module, the number of hidden units is n∗k, which is the number of activities multiplied by the *k*-shot. The input size of Bi-LSTM is decided by the dimension of a fully-connected layer which is 64. The number of hidden layers is 1. Hidden size (the dimension of the hidden layer) is 32, while, in attLSTM, it is 64. We minimize the cross-entropy loss function with Adam to optimize the model parameters. The exponential decay rate ρ1 and ρ2 are empirically set as 0.9 and 0.999. The learning rate is set as 0.0001. The total number of training iterations is 300. The batch size is set as 16. Unless otherwise specified, the following evaluations follow the above settings.

### 4.2. Overall Performance

**Figure 13:**
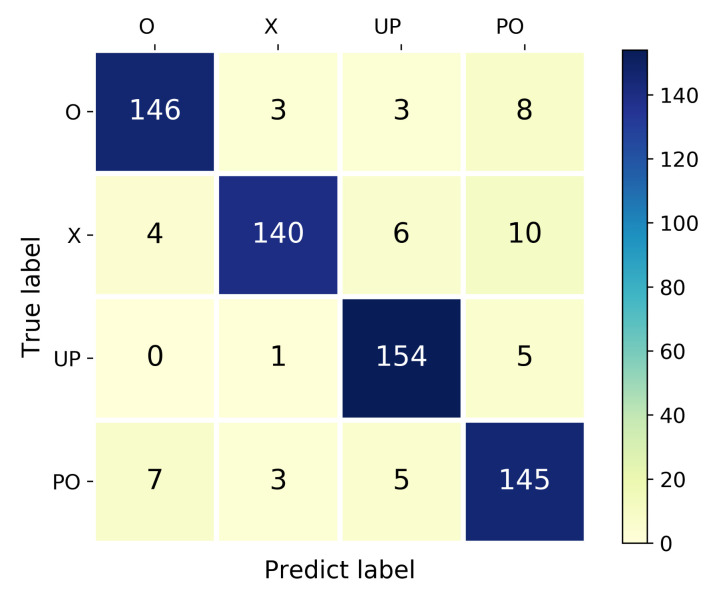
The confusion matrix of location-independent human activity recognition.

Table 2 illustrates the recognition average accuracy of our method compared with the traditional deep learning method CNN and WiHand [25]. WiHand is based on the low rank and sparse decomposition (LRSD) algorithm and extracts the histogram of the gesture CSI profile as the features, which outperforms the other location-independent approach. It can be seen that our system outperforms these two methods in both location-dependent sensing and location-independent sensing. All the methods can recognize with high accuracy for single location sensing and mixed locations sensing. For the location-independent sensing, WiLiMetaSensing can also obtain an average 91.11% recognition accuracy, which is about 7% higher than CNN, and about 9% higher than WiHand. Specifically, the confusion matrix of a test for our location-independent human activity recognition method is shown in Figure 13 with a 91.41% accuracy. We can see that all of the activities can be recognized with high accuracy. Note that Table 2 shows the optimal recognition accuracy of WiHand with 30 subcarriers and 20 features. We analyze the reason why WiHand did not perform as well as the original dataset, including (1) The nine data collection locations of WiHand are relatively close to the TX and RX, while our 24 locations have a wider coverage. (2) The sampling rate of WiHand is 2500 packets/s, which is much larger than our 200 packets/s. (3) WiHand could extract CSI streams of all 56 subcarriers from the customized drivers, while ours is 30 subcarriers. A higher sampling rate and more subcarriers may provide richer fine-grained information. After the matrix decomposition, more activity-related information will be preserved.

### 4.3. Module Study

Comparison with different feature representation modules. In this section, we explore the effect of the embedding module *g* (Bi-LSTM) and *f* (attLSTM) for the samples from the support set and the query set. We test for one-shot learning using the samples with 90 subcarriers. From Table 3, we can see that both modules enhance the performance of the method. Leveraging all the activity samples from the support set, common features can be obtained to adjust the feature representation, so as to pay more attention to the location-independent features. The embedding module for the query set enables the sample in the source domain to effectively calibrate the feature representation of the sample in the target domain.

### 4.4. Robustness Evaluation

**Figure 14:**
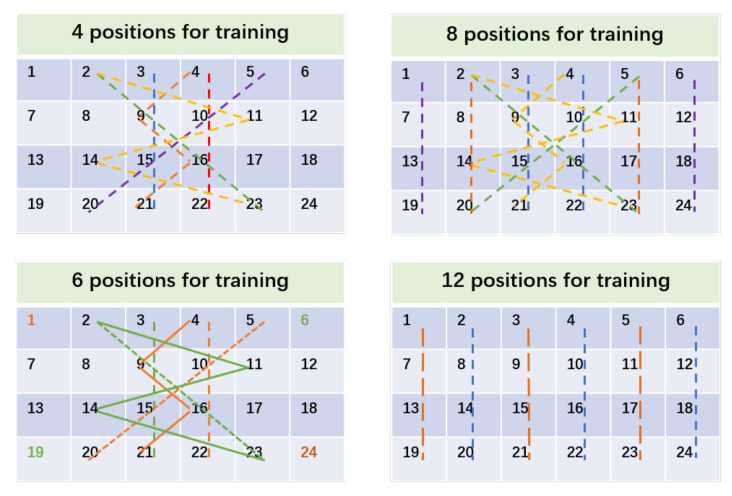
The layout of the training locations.

Performance of location-independent sensing in terms of different number of training locations. The activity samples of each position have different data distributions. The further the distance of the locations, the higher the probability of a broader distribution distance will be. Therefore, when it comes to the samples collected for training the models, we hope the positions of the training samples become more decentralized. We adopt a fixed training position selection strategy, in which the positions should be distributed as far as possible in the entire space, instead of clustering together in a line parallel to the transceiver. We choose 4/6/8/12/24 locations for training and 24 locations for testing. The selections of fixed 4/6/8/12 training positions are depicted in Figure 14. Specifically, for 4/8/12 training locations, the positions where the same colored straight line goes through, or the inflection points and the enthesis of the same colored broken lines, constitute the training samples. For six training locations, the straight lines or broken lines together with the same colored marked locations form the training pairs.

As demonstrated in Table 4, when we pick four training locations and 1-shot, the accuracy is 91.11%. When eight training locations and 1-shot are selected, the accuracy is 92.66%. The results indicate that the more training positions there are, the higher accuracy the recognition obtains.

Performance of location-independent sensing for samples with different numbers of subcarriers. We explore one-shot human activity recognition with different numbers of subcarriers. As illustrated in Table 5, the recognition accuracy reduces with the decrease of the number of subcarriers. However, it still maintains an acceptable recognition rate when there are only 30 subcarriers from one pair of antenna.

Performance of location-independent sensing for different TX-RX antenna pairs. We investigate the recognition accuracy with 30 subcarriers from different TX-RX antennas. As shown in Table 6, different antenna pairs have similar recognition effects. The difference reflects that different antenna pairs contain more or less diverse information. Therefore, 90 subcarriers which integrate these features can obtain superior results. Note that, in Table 6, *i*TX-*j*RX represents CSI data from *i*-th TX and *j*-th RX.

Performance of location-independent sensing for different number of shots. We explore the number of samples in support set for testing. As examples, we also select four locations for training and 24 locations for testing. The samples with 90 subcarriers are used. The identification results are listed in Table 7. It is noted that all the average accuracy is above 90%, and the accuracy will increase with the growth of the sample size.

**Figure 15:**
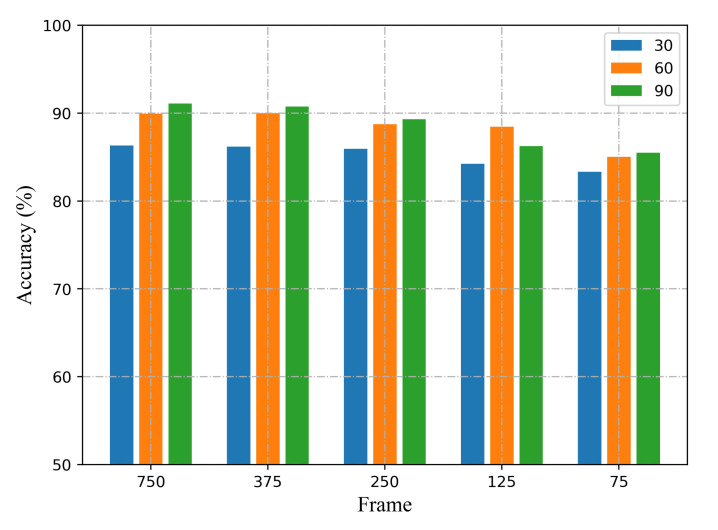
The recognition accuracy with different number of subcarriers and sampling rates.

Performance of location-independent sensing for samples with different sampling rates. We collect CSI measurements at the initial transmission rate of 200 packets/s, and down-sample the 750 CSI series to 375, 250, 150, 75. The one-shot results with different sampling rates are shown in Figure 15. As can be seen, when he sampling rate decrease to 20 frames/s, the method can still obtain satisfying accuracy.

## 5. Limitations and Future Work

Although the proposed WiLiMetaSensing system realizes location-independent sensing with very few samples, there remain many challenges to be overcome. First of all, there are some strict restrictions in the data collection process. For example, volunteers are required to perform the same activity facing nearly the same direction in the same room. Consequently, except for the types of activity and location variations, other factors that would affect the transmission of the signals are not seriously taken into account, such as the status of a person (e.g., pose and direction) and the environmental variations (i.e., altering the room or the locations of surrounding objects). In addition, as signals can be easily blocked, reflected, or scattered by different targets, the existence of other people would also result in different signal patterns. However, the impact of interference from the other person on the classification accuracy was either not considered. As a result, in future work, we will further explore the generalized and robust human activity recognition method with an adequate account of these aforementioned factors. Only in this way can human activity recognition technology develop from academic research to industrial application.

## 6. Conclusions

In this paper, we present a novel human activity recognition system, named WiLiMetaSensing. It realizes location-independent sensing with very few samples in the Wi-Fi environment. Inspired by the idea of meta learning, we endow the system with the ability that can utilize the knowledge acquired from one location for others. Technically, we propose a CNN-LSTM feature representation and metric learning-based human activity recognition system. The model focuses on the common characteristics of different locations and extracts discriminative features for different activities. The performance evaluation is conducted on the comprehensive dataset we build. It demonstrates that the WiLiMetaSensing system can achieve an average accuracy of 91.11%, with four locations for training, given only one sample for other testing locations. More importantly, it can well adapt to the data samples with a small number of subcarriers and a low sampling rate. Therefore, we can firmly conclude that the presented approach is feasible and robust for location-independent sensing.

## Figures and Tables

**Table 1:** Predefined activities.

Mark	Activity
O	Draw a circle with right hand
X	Draw a cross with right hand
PO	Lift up and lay down two arms
UP	Push and open with two arms

**Table 2:** The recognition accuracy for single location sensing, mixed locations sensing, and location-independent sensing.

Accuracy (%)	WiLiMetaSensing	CNN	WiHand [26]
Single location	99.13	99.00	96.15
Mixed locations	98.36	95.53	91.50
Location-independent	91.11	84.02	82.20

**Table 3:** The recognition accuracy with different embedding modules.

Embedding Modules	Accuracy (%)
WiLiMetaSensing with Bi-LSTM with attLSTM	91.11
WiLiMetaSensing with Bi-LSTM without attLSTM	90.25
WiLiMetaSensing without Bi-LSTM without attLSTM	88.73

**Table 4:** The average recognition accuracy for different numbers of training locations.

Number of Training Locations	4	6	8	12	24
Accuracy (%)	91.11	92.23	94.98	96.00	98.36

**Table 5:** The accuracy for different number of subcarriers with four training locations.

Training Locations	90 Subcarriers	60 Subcarriers	30 Subcarriers
3,8,15,20	92.63	91.75	86.75
1,10,13,22	91.25	88.50	87.50
2,8,14,20	91.75	90.75	87.75
3,9,15,21	89.13	89.00	85.13
1,8,15,22	91.25	89.50	85.75
4,9,14,19	90.63	90.13	85.00
Average accuracy (%)	91.11	89.94	86.31

**Table 6:** The accuracy for different TX-RX antenna pairs.

TX-RX	1TX-1RX	1TX-2RX	1TX-3RX
Accuracy (%)	86.31	87.00	85.60

**Table 7:** The accuracy for different number of shots with four training locations.

Number of Shots	1-Shot	2-Shot	3-Shot
Accuracy (%)	91.11	92.25	93.21

## Data Availability

Not applicable.
